# Pilot study of quality of care training and knowledge in Sub-Saharan African medical schools

**DOI:** 10.5116/ijme.595b.b38c

**Published:** 2017-07-24

**Authors:** Diana Bowser, Yasmin Abbas, Temitope Odunleye, Edward Broughton, Thomas Bossert

**Affiliations:** 1 Global Health Policy and Management, Heller School for Social Policy and Management, Brandeis University, USA; 2 Roche Pharmaceuticals, Egypt; 3 Heller School for Social Policy and Management, Brandeis University, USA; 4 Johns Hopkins School of Public Health, University Research Company, LLC, USA; 5 Harvard T.H. Chan School of Public Health, USA

**Keywords:** Quality of care, medical schools, sub-Saharan Africa, medical errors, healthcare improvement

## Abstract

**Objectives:**

To identify the level of knowledge and competencies related to quality of care during medical education in sub-Saharan African medical schools.

**Methods:**

A cross-sectional study design was utilized to examine the capacity of medical schools in sub-Saharan African (SSA) countries to teach about the concepts of quality of care and the inclusion of these concepts in their curriculum. A purposeful convenience sampling technique was used to select participants from 25 medical schools in 5 sub-Saharan African countries. Respondents included medical school deans or senior academic personnel.  A survey was developed using the Institute of Medicine’s definition of quality of care as the guiding framework.  Sample means and summary statistics were used to present the results of the survey responses.

**Results:**

While 45% of the schools surveyed are teaching on at least one of the six domains of the Institute of Medicine’s definition of quality of care, there are some schools who report not teaching about quality at all, or that they “do not know”. Despite these low numbers, when asked about topics related to quality of care, many schools are teaching applied management related topics and almost all schools teach about equity and patient-centered care.

**Conclusions:**

The results have important impacts both for incorporating quality of care into medical education and for practitioners.  The tool developed for this study can be used in future qualitative and quantitative studies to further understanding of how to improve the teaching and learning about quality of care in medical schools. Keywords: quality of care, medical schools, sub-Saharan Africa, medical errors, healthcare improvement

## Introduction

Quality of care is an important aspect of delivering effective healthcare services and a critical feature of access to care, especially in sub-Saharan Africa where emphasis on densities and distribution of health workers often ignores measures to improve the quality of the care.^1^^-^^3^  While teaching about quality of care can be addressed at both the point of service and during pre-service medical education, it is unclear at which stage it is most frequently taught in sub-Saharan Africa.^4^  To ensure all medical school graduates have a basic understanding of issues of quality of care and how it can be addressed in practice, it is important to include it in standard pre-service education curricula.^5^^,^^6^ In addition, the understanding of the concept of quality can also vary.  While some health care institutions embrace a broader definition, such as that supported by the Institute of Medicine, others focus more closely on certain aspects of quality such as effectiveness and safety.^7^^,^^8^ 

One of the most important studies to date that has examined medical education in sub-Saharan Africa is that of Mullan et al. The study examined key aspects of the health systems in each country and how this impacts medical education, as well as shortages in medical school facilities and weaknesses in the infrastructure.  While Mullan et al. reference the importance of quality, they mainly link this concept with accreditation of facilities and do not report in detail the level of quality that is taught within medical institutions.^9^ Several other studies have also examined different aspects of the medical system in sub-Saharan Africa with minimal focus on quality of care.  Greysen et al., for example, highlights the need for more development of medical education as a “specialized field of inquiry” focusing on the four main weaknesses in medical education in Sub-Saharan Africa: technology, financing for medical education, increasing the number of human resources for health and post graduate training, and primary care.^10^ Furthermore, Nangami et al. find that while schools of public health in eastern and central African countries teach courses that are relevant to health systems strengthening within master’s level programs, there is limited capacity to teach about health policy, including quality, due to outdated curricula, staff competency constraints, and limited access to resources.^11^

While these studies have addressed general issues of medical education, there are clear gaps in the literature about knowledge and competency related to health care quality improvement in medical schools in Sub-Saharan Africa.  To begin to address this gap, this study developed a study design, interview tool and conducted interviews with deans of medical schools in sub-Saharan Africa.  Using results from a WHO study that examined medical school training more generally in sub-Saharan Africa,^9^ a similar framework is applied and expanded to examine how quality of care is taught within a broader medical school curriculum. The expanded framework focuses on the capacity, teaching style, and methods used to teach about quality of care using the Institute of Medicine’s definition of quality of care.

The Institute of Medicine’s definition of quality of care focuses on the following six domains of systems in order to reduce illness, injury, and disability and to improve population health: effectiveness, efficiency, timely care, patient safety, equitable care, and patient-centered care.^12^ Effective and reliable care emphasizes that health care services should only be based on scientific evidence and knowledge. Preventing harm to the patients, which could either be through errors or risky delayed services, requires focus on patient safety and availability of timely services. The appropriate use of resources, through reducing waste (technical efficiency) and proper allocation of such resources, would ensure efficiency of care.^13^ Equitable care means that all patients could receive the needed care, regardless of their age, gender, or geographical location. Lastly, patient-centered care denotes that the system is respectful to patient needs, values and preferences. This means that no patient shall receive care against their values or beliefs.^12^ The Institute of Medicine’s confirms that the health care system that realizes those six domains is more capable of meeting patients’ needs and achieving the desired health outcomes of individuals and the population it serves.

Using this framework, the aim of the study is to identify training concepts related to quality of care that are taught during pre-service medical education and post-graduate continuing medical education in sub-Saharan African medical schools. This is the first study of its kind to measure the actual level of knowledge and competencies related to quality of care in medical school curricula in sub-Saharan Africa.  The study focuses on the following four questions:

   •   Do medical schools in sub-Saharan Africa teach about quality of care/health care improvement?

   •   What is the capacity of medical schools to teach about quality of care/health care improvement?

   •   How do medical schools (i.e. teaching style used) teach about quality of care/health care improvement?

   •   What are the macro health system policies in place (at the school or country level) to promote teaching of quality of care/health care improvement and eventually health outcomes?

## Methods

### Study design and participants

This pilot cross-sectional study was designed to examine the capacity of medical schools in sub-Saharan African (SSA) countries to teach about the concepts of quality of care and the inclusion of these concepts in their curricula. The Institute of Medicine’s definition of quality of care was used as the guiding framework for the study.  The Institute of Medicine’s definition incorporated the following six domains as part of their definition of quality: effective care, efficient care, timely care, patient safety, equitable care, and patient-centered care.

A thorough literature review was conducted on what constitutes knowledge and competencies for health care quality improvement in a typical medical school in sub-Saharan Africa. Thereafter, a tool to assess what was being taught in pre-service training on this topic in SSA medical schools was developed. This was a comprehensive self-administered, structured questionnaire with 30 questions covering six sections:  participant characteristics, school characteristics, training, knowledge of quality of care, knowledge of healthcare management skills, and equity and patients’ rights. The survey instruments were prepared in English and translated to French for the participants from the Democratic Republic of Congo. The questionnaires, along with the consent forms, were electronically sent to the prospective respondents by e-mail. Signed consent forms and filled questionnaires were sent back electronically to the research team.

Participants were selected using a purposeful convenience sampling focusing on countries from sub-Saharan African. Countries that had schools of medicine and ASSIST Project offices were ranked according to their Gross Domestic Product (GDP) per capita and life expectancy at birth for the year 2014, as indicators of economic prosperity and the strength of the health system in each of the countries. Countries were chosen to ensure variation across both GDP per capita and life expectancy (low, medium and high).

A total of 12 countries with ASSIST offices were identified.  Five of these countries were selected based on variation in GDP/capita and life expectancy. The sample included one medical school from Botswana, eight medical schools from South Africa, five medical schools from Uganda, five medical schools from Tanzania, and nine medical schools from Democratic Republic of Congo (DRC), for a total of 28 medical schools. However, due to lack of contact details and limited communication, the following three medical schools were dropped from the analysis:  the medical school in Botswana, one medical school in DRC, and one medical school in South Africa. The final sample consisted of a total of 25 medical schools in four countries.

The intended respondents were senior academic personnel including the deans of the medical schools, as well as some heads-of department of medicine, surgery, pediatrics and obstetrics/gynecology. No personal information was asked of these senior academics, rather only publicly available data on the details of the medical curriculum.

A protocol and informed consent were developed and submitted to the Harvard T.H. Chan School of Public Health’s IRB for expedited review.  Based on the IRB review, the study was deemed exempt because the Research Information Security Level was classified, using Harvard’s Data Security Policy, as the lowest level, Level 1.   The questionnaires were accompanied by a written consent form which participants signed prior to taking the survey. Confidentiality was strictly ensured and all gathered questionnaires were securely archived.

### Data collection methods

Using the Institute of Medicine’s definition as the guiding framework, a questionnaire was developed to understand the level and capacity of medical schools to teach about the concept of quality of care.  The questionnaire was designed to understand and triangulate how each medical school incorporated each of the six aspects of quality in the Institute of Medicine’s definition into their medical school curriculum.  All six aspects of quality were explored further in the questionnaire from a management and equity lens. Patient safety was examined in more detail to understand the overlap between quality and medical errors, based on the gap in this area globally.^12^^,^^14^ The survey was validated through the use of a similar framework and set of questions that were utilized in the WHO study about medical education in Sub-Saharan African countries.^9^  The survey utilized in the WHO survey was validated and tested and utilized in 146 medical schools.

The questionnaire had 32 questions in total, some of which were divided into sub-questions.  All questions were either yes/no, categorical, or open ended.  All questions were grouped into five main areas for analysis: basic demographic/environmental information, capacity to teach about quality, teaching style, practices used to teach about quality, and macro health policies in place to promote teaching about quality of care. All questions were equally weighted, with no scores assigned to the questions. Responses to the questions were analyzed accordingly (see data analysis below). The response rate for the survey is 44%.  Of the 25 questionnaires sent to the participating schools, 11 responses were received.

### Procedure

Surveys were sent via email to the deans of the participating schools (N=25), who asked to answer the questionnaire themselves and also to send the survey to the four heads of their main medical school departments (medicine, surgery, pediatrics and obstetrics/gynecology).  Follow up phone calls were made if participants did not respond to email inquiry within two weeks.  Surveys were collected via email from responding participants.   

### Data analysis

Data collected through the questionnaires were extracted into an Excel spread sheet for coding and analysis.  Data were then transferred to data analytical software (STATA).  The answers to the survey tool were used to summarize the responses for the final medical schools in the sample in each of the main areas of the survey:  number of schools that teach about quality of care using the Institute of Medicine’s definition, capacity to teach about quality of care, methodologies used to teach about quality of care, and macro health policies in place to promote teaching of quality of care.  Due to the small sample size, sample means for relevant questions and summary totals were used to present the results of the survey responses.

## Results

### Characteristics of medical schools

Table 1 highlights the basic characteristics of the 11 medical schools that responded to the survey.  As shown in Table 1, there are five respondents from South Africa, one from Uganda, two from DRC and three from Tanzania. On average, the schools included in the study have been in existence for 33 years and students can obtain their basic medical degree after about six years of study. All the schools use English as the language of instruction, except the DRC schools where French is used. There is minimal variation with regard to the other measured characteristics of the schools. All schools are affiliated with a university or hospital and schools in all countries except Tanzania are publicly owned.

**Table 1:** Summary statistics of facilities by country

Country	Total surveys	Age of school Mean	Length of study Mean	Taught in English^*^ number/n	Publicly owned number/n	University affiliated number/n	Hospital affiliated number/n
South Africa	5^**^	49	6	4/4	4/4	4/4	4/4
Uganda	1	12	5	1/1	1/1	1/1	1/1
DRC	2	40	7	0/0	2/2	2/2	2/2
Tanzania	3	14	5	3/3	2/3	3/3	3/3
Botswana	0	---	---	---	--	--	--
Average (Total)	11	33	6	8/10	9/10	10/10	10/10

*In comparison to French and other languages;

**One South African survey had a number of missing variables resulting in n=4 for many responses

The survey results also show that a number of education programs are available in the schools surveyed. The most common programs are medicine, public health and nursing, where almost all of schools surveyed have courses in these disciplines. Dentistry is taught only in the Tanzanian schools.  A pharmacy program is available in half of the schools in DRC, in the one school in Uganda and two of the three Tanzanian schools. Health care management is taught in two schools in South Africa, one school in DRC and two schools in Tanzania. Other education programs taught in some of the schools include community health, laboratory medicine, physiotherapy, occupational therapy, social work.

### Level of teaching about quality of care

Table 2 shows the survey results for general and applied topics related to quality of care.  The first row of Table 2 shows that overall while 45% (five out of the eleven schools) report teaching about at least one aspect of quality of care, at least half of the schools in South Africa, DRC and Tanzania report not teaching about any aspects of quality or report that they “do not know”.  

To better understand the participants’ understanding of the elements of quality of care, according to the definition by Institute of Medicine, and their application in practice, the survey also examined if specific quality of care topics are taught in each medical school.  The results are summarized in the lower part of Table 2.  Each of the topic areas relates to a specific aspect of the Institute of Medicine’s domains of quality, some focusing more on management issues and other areas focusing more on equity and patient rights.  The Ugandan school reported teaching on all six management related aspects of quality (health care management, use of resources, patient flow, waiting time, follow up care, and payment and reimbursement) as well as all equity related topics.  There is more variation in the level of teaching of these topics across the schools in the other study countries.  The topics taught most frequently are the equity related topics, how to manage a health care facility and efficient use of resources (i.e. how to best use available resources).

Due to the importance of understanding and reducing medical errors, three concepts of medical errors taught were also assessed through the survey: (i) harmful consequences of delayed care, (ii) reporting errors and (iii) avoiding and handling errors. Generally, all the schools include teaching about medical errors in their medical training. Almost all, approximately 80% of the schools, teach about reporting errors and avoiding and handling errors (four out of the five South African schools, two out of the three Tanzanian schools, the two DRC schools, and the Ugandan school). However, while the other schools also teach about harmful consequences of delayed care, the DRC schools did not report teaching this concept.

**Table 2:** General and applied topics of quality of care taught

Country	South Africa	Uganda	DRC	Tanzania	Mean
At least one aspect of quality	2/5	1/1	1/2	1/3	5/11
Management related aspects of quality					
Efficiency of resources	3/5	1/1	1/2	2/3	7/11
Management of healthcare facilities	2/5	1/1	1/2	3/3	7/11
Patient flow	2/5	1/1	1/2	1/3	5/11
Timing of follow up care	2/5	1/1	0/0	2/3	5/11
In-facility waiting time	1/5	1/1	1/2	1/3	4/11
Payments and reimbursement schemes	0/0	1/1	0/0	0/3	1/11
Equity related aspects of quality					
Healthcare equity	5/5	1/1	2/2	2/3	10/11
Services for vulnerable groups	5/5	1/1	2/2	2/3	10/11
Patients’ rights	5/5	1/1	2/2	2/3	10/11
Patients’ values and needs	4/5	1/1	1/2	2/3	8/11

*Number of schools out of total schools

### Capacity to teach about quality of care

The results related to capacity to teach about quality of care focus on the type of class used to teach about quality of care, when quality is taught, and who teaches the quality topics.  The two schools reporting that quality is not taught confirm that there is no specified class to teach these concepts.  The four schools that report they do not know if quality is taught in the medical school also report that they do not know how these concepts are taught.  Finally, for the four schools that confirm that quality topics are taught, report that these concepts on not taught in a standalone course. 

Medical school curriculum is traditionally divided into theoretical “pre-clinical” training followed by more practice “clinical” training. In order to understand at which stage each medical school could incorporate additional quality of care training within these traditional stages of training, respondents were asked at which stage quality of care was taught. The six Institute of Medicine domains of quality of care are taught to medical students and/or professionals at different points during their training; either clinically/during residency or theoretically/during internship. The results show that in the one Ugandan school, all six concepts are taught both clinically and theoretically. In South Africa, slightly more of the concepts are taught clinically (46%) than theoretically (21%). In Tanzania, 33% of the concepts are taught both clinically and theoretically.

Results show that where quality of care is taught, it is taught by professors of the same school in most of the schools in South Africa, Tanzania, and in one of the schools in DRC. However, it is taught by an external professor in the Ugandan school.

### Teaching style related to quality of care

In order to have a better understanding of the framework within which medical schools in Sub-Saharan Africa teach about quality of care, and whether they could benefit from developing a more structured framework for teaching about quality of care, the survey asked respondents about the following three different teaching styles that could be utilized in the different training stages: 1) community-based learning - a form of instruction where trainees learn professional competencies in a community setting, focusing on population groups and individuals, and their day-to-day problems, 2) multi-disciplinary team-based learning - an instructional approach aimed at preparing students to effectively work within healthcare teams that include other health professionals, and 3) patient feedback - a structural channel for patients and their families to express their treatment preferences and concerns. The results show that there is no noticeable pattern to how medical schools are utilizing these three different teaching styles in their “pre-clinical” and “clinical” training. The South African schools demonstrate the most variation, showing that their medical schools use all three forms of instruction (community-based, multi-disciplinary team-based and patient feedback) across all learning stages (preclinical courses and clinical courses).  The one medical school from Uganda reported using preclinical courses for community based learning and both preclinical and clinical rotations for multidisciplinary learning and incorporating patient feedback into their medical school learning. The schools from DRC and Tanzania report more of a mixed combination of preclinical and clinical rotations across the three teaching styles.

### Health systems and quality of care

The survey also asks about additional health system indicators that might strengthen the curriculum and learning around quality of care. For example, there are a number of quality management teams present in the different hospitals affiliated with the medical schools included in the study. However, it was not clear from the responses whether these teams contribute to the training of students during their clinical training stage. There are also different levels of examinations after medical school that test for additional skills, specialization and other skills that are related to quality of care. 

In all the schools, internship is a compulsory requirement for licensed medical practice. In one of the Tanzanian schools, postgraduate studies and community service are additional requirements. Community service is also an additional requirement in all South African schools. The practice of quality of care through direct patient care is carried out in the Ugandan school, one of the South African schools, two of the DRC, and two of the Tanzanian schools.

## Discussion

This is the first pilot study to design a survey to understand different aspects of how quality of care is taught in medical schools, focusing on sub-Saharan Africa. The study implemented the survey in 25 medical schools across five countries in sub-Saharan Africa and calculated the level of knowledge about teaching on quality of care from survey respondents.

Despite the small sample size, the results of this pilot study are important for beginning to understand the first research question, related to the baseline level of teaching on quality of care and health care improvement in medical schools in Sub-Saharan Africa.  The results demonstrate that the level of teaching on  quality of care is rather low; with just under 50% of survey respondents reporting that they teach about at least one aspect of quality of care as well as a number of medical schools reporting to “not know” if quality of care is part of the medical school curriculum. These results show that there is room for not only improvement in current quality of care pedagogy, but also a broader understanding of how to develop such initiatives in medical schools in sub-Saharan Africa.  Focusing training initiatives in medical schools could potentially be a powerful platform to increase knowledge on basic understanding of quality of care and health care improvement. This would not only increase the general level of awareness of concepts of quality and health care improvement, but also allow medical schools to become key players in addressing the quality deficiencies in their countries. Incorporating formal teaching on quality of care in medical schools provides an expansive platform to ensure that future physicians learn about quality improvement, how to think about continuously incorporating quality initiatives into their work, and ultimately improve quality of care and patient outcomes.^4^^,^^15^

In addition to the low level of reported teaching on quality of care in medical schools included in this study, the results related to the second and third research questions (type of class used for teaching as well as the capacity to teach on quality of care) are worth noting. No medical school that reported teaching about quality of care offers a standalone class on quality. These results suggest that quality topics, if taught, are interspersed into regular theoretical and clinical courses. Not having a standalone course on quality does not allow for a thorough theoretical explanation of quality of care or a deeper understanding of issues surrounding measurement, quality improvement, problem identification, and the role of the patient and provider in improving quality. The results related to who teaches the quality courses suggest that the same professors teaching the theoretical and clinical courses are the ones who are adding pieces of quality into their theoretical and clinical course offerings. These trends highlight a critical shortage of not only practising human resources for health, but also medical professors to teach more topics in medical schools.^16^ Overall, the results indicate the lack of clear framework and methodology to teach about quality of care in medical school in Sub-Saharan Africa. Even among those schools who report teaching about quality of care, there is variation with regard to capacity, stage at which quality is taught and style of teaching. Due to this variability, it would be difficult to gauge the how quality of care principles are being practiced among medical graduates, even within the same country.

The final research question examined the relationship between the broader health system in each country and quality of care. The results from the study show the potential importance of quality management teams, hospital policies on quality as well as incorporating quality training into licensure and re-licensure. Each of these areas is important to investigate in future studies. Many other system areas also need to be incorporated into a larger study including financing and payment policies, number of human resources as well as the organization of the system.  

As mentioned above, a particular innovation of this study is how the survey applies the following categories of curriculum assessment to examining how quality of care is taught in a particular region of the world: characteristics of medical schools included in the study, level of teaching about quality of care, capacity to teach about quality of care, and relationship to country health systems. The framework developed in this survey is helpful in analyzing the results and in assisting countries as they begin to think about expanding their teaching and learning on quality of care using the Institute of Medicine’s definition.  In addition to teaching about pieces of quality throughout pre-clinical and clinical courses, medical schools need to introduce medical students to a broader framework of quality and how to apply this in their future careers.  

The only other study that has attempted to measure pedagogy and training around quality of care in medical schools in sub-Saharan Africa is a WHO study that measured 13 different factors related to medical education in Africa with a sample of 105 medical schools.  A small section of this survey focuses on accreditation and quality assurance.^9^ This survey builds on the WHO work in several ways in order to understand in more detail how quality of care is taught in medical schools.  First, the survey used in this study utilizes a broad definition of quality of care based on the Institute of Medicine’s definition.  Second, the survey asks respondents directly about their understanding of this definition of quality of care and also triangulates this understanding with additional questions about specific teaching practices related to aspects of management of health care services, equity principles, and patient-centered care.  Finally, the survey attempts to understand not only how schools teach about quality of care, but also the capacity to teach, methods of teaching and link to macro health policies.

The greatest limitation to this research is the difficulty in data collection and survey response rate.  The original methodology was sent to survey deans of all medical schools as well as heads of departments.  These results would have provided an excellent database on baseline levels of teaching of quality of care. The low response rate substantively limited the analysis presented above. The low response rate may be a reflection of the pressure and workload these medical schools in sub-Saharan Africa are under.  The next phase of this research should consider alternative methods to gathering this important data such as questionnaires administered in person by research assistants.  In addition, quality of care needs to embrace a true team effort and additional research needs to be conducted to understand how these concepts are also taught in nursing schools, pharmaceutical schools and schools of public health.

## Conclusions

This is a pilot study examining how quality of care is taught in medical schools in sub-Saharan Africa. The results suggest an insufficient amount of attention to the topic and that the magnitude of this gap deserves additional research.  For medical education, the results show that initiatives in this area need to focus not just on curriculum, but the capacity of schools and faculty to train in this area. The results underlie a larger call for action on developing and improving teaching about quality of care in medical schools in SSA. For practitioners, a course on quality of care with applied learning is imperative at some point during or after medical school training. This ultimately will improve quality of health care services and patient outcomes. The tool developed for this study can be used in future studies to expand the sample to ascertain a better understanding of how to improve the teaching and learning about quality of care.

### Acknowledgements

This study was funded through a grant to the Harvard T.H. Chan School of Public Health from University Research Co., LLC (URC) under the USAID Applying Science to Strengthen and Improve Systems (ASSIST) Project, which is made possible by the support of the American people through the United States Agency for International Development (USAID).  The USAID ASSIST Project is managed by URC under Cooperative Agreement Number AID-OAA-A-12-00101.  The views expressed are those of the authors and do not necessarily reflect the views of USAID or the United States Government.  ASSIST is the primary funder of this activity.

### Conflict of Interest

The authors declare that they have no conflict of interest.
